# Cancer Survivors' Experiences With Six Months of Community Health Worker Health‐Related Social Needs Support: A Qualitative Analysis of Perceived Impact

**DOI:** 10.1002/pon.70649

**Published:** 2026-11-05

**Authors:** Arianna Milicia, Marjanna Smith, Jessica Rivera Rivera, Mandi L. Pratt‐Chapman, Kimberly Davis, Hannah Arem

**Affiliations:** ^1^ Implementation Science MedStar Health Research Institute Columbia Maryland USA; ^2^ MedStar Health National Center for Human Factors in Healthcare MedStar Health Research Institute Washington Columbia USA; ^3^ Department of Medicine at George Washington University School of Medicine Washington Columbia USA; ^4^ Department of Oncology Georgetown University Washington Columbia USA

**Keywords:** cancer, cancer survivorship, community health workers, health‐related social needs, oncology, quality of life, social support

## Abstract

**Background:**

Cancer survivors face persistent social, emotional, and practical challenges that can worsen health and quality of life. These unmet needs disproportionately affect Black cancer survivors and contribute to inequitable outcomes.

**Aims:**

This qualitative study explores the experiences and preferences of Black cancer survivors participating in a community health worker (CHW) supported intervention to assess and address health‐related social needs (HRSNs).

**Methods:**

Semi‐structured interviews were conducted with participants enrolled in a 6‐month CHW intervention in the United States to assess and address social needs. A mix of inductive and deductive coding was used within a constructivist framework to identify themes aligning with Patient‐Reported Outcomes Measurement Information System (PROMIS) social support and quality of life (QOL) domains.

**Results:**

Thirty interviews were conducted. Most participants were female (*n* = 22), had breast cancer (*n* = 14), and experienced social needs (*n* = 22). Participants described significant emotional, practical, and financial challenges during survivorship. Participants commonly cited that CHWs provided significant emotional support, as well as food and transportation referrals. Over half of participants reported increased awareness and self‐efficacy in navigating community resources. Most participants reported improvements in quality of life, particularly with enhanced emotional well‐being and instrumental support, that were vital to their daily functioning.

**Conclusion:**

Consistent, monthly phone‐based CHW support improved emotional well‐being, self‐efficacy, and overall quality of life. Future research should explore the impact of interventions on psychometrically validated quality of life measures and receipt of appropriate survivorship care.

## 1 Background

The population of cancer survivors in the United States was estimated at 18.6 million in 2025, with a projected increase to 26 million by 2040 [1]. A growing older population and advancements in early detection and treatment have increased cancer survival rates in the United States [1]. A cancer diagnosis and treatment can cause or exacerbate health‐related social needs (HRSNs) such as housing, food security, transportation, emotional support, and financial stability [2].

In the United States, Black populations have a high burden of unmet HRSNs, which are well‐documented drivers of racial inequities and contribute to disproportionately worse cancer outcomes [3, 4]. Unmet social needs contribute to these adverse outcomes and increased healthcare utilization [5].

Unaddressed physical, cognitive, psychological, emotional, and financial challenges during cancer survivorship can have longer‐term health detriments [1, 6]. Studies have shown that 25% of cancer survivors have persistent physical and mental problems, including anxiety, depression, and other psychological and social distress [7, 8]. HRSNs are further associated with higher prevalence of chronic conditions, depressive symptoms, perceived stress, and poorer overall physical and mental health [5, 9]. These unmet needs have also been linked to adverse health‐related behaviors, including tobacco use, inadequate nutrition, physical activity, insufficient sleep, and lower engagement in preventative care [5, 9, 10, 11]. Addressing HRSNs among cancer survivors may facilitate healthier behavioral patterns, improve disease management, and promote sustained well‐being [12].

Social support is particularly critical, as survivors may struggle with emotional and physical challenges, including feelings of vulnerability, depression, sadness, and anxiety [7, 8, 13]. According to the National Cancer Institute, social support is a network of family members, friends, neighbors, and community members who provide cancer patients with psychological, physical, and financial support when they need it [14]. Cancer patients who have greater social support demonstrate higher quality of life and lower mortality risk, in addition to improvements in physical health, emotional well‐being, and overall survival [15]. Different types of social support that an individual may experience include emotional support, informational support, esteem support, and instrumental support, which are essential for survivors navigating post‐treatment challenges and maintaining overall well‐being [8, 13].

Theoretical models linking social support to physical health suggest that supportive relationships may influence health outcomes through psychological and behavioral mechanisms [16]. However, few studies have been published that focus on cancer survivors' experiences with social needs support and how this impacts their quality of life, particularly among Black populations [3, 17]. In this qualitative study, we sought to understand Black cancer survivors' experiences and perceived impact of receiving 6 months of community health worker (CHW) support to address HRSNs.

## 2 Methods

### 2.1 Research Method and Study Design

Guided by a constructivist qualitative framework [18], this study analyzed participants' lived experiences and perceived impacts of CHW support, with mechanisms of action framed through Uchino et al.‘s [16] model linking social support to physical health. This model outlines how structural and functional aspects of social support influence health outcomes through psychological (e.g., depression, stress reduction, emotional well‐being, quality of life) and behavioral (e.g., health behaviors) pathways. This framework provided a conceptual lens for interpreting how emotional, instrumental, informational, and psychosocial supports from CHWs were perceived to affect survivors' quality of life and daily functioning.

Consistent with this framework, we used thematic analysis to identify patterns in participants' narratives describing how CHW social support influenced their emotional well‐being, ability to navigate resources, health‐related behaviors, and perceived quality of life [19].

### 2.2 Study Sample

Interviews were conducted as part of parent study, Building Equal Access Together in Cancer (BEAT‐C) [20, 21, 22]. Adult (age 18+) cancer survivors were recruited from two health systems in the greater Washington DC/Baltimore region of the United States (4 hospitals) that serve large proportions of socio‐economically disadvantaged and racial minority patients. In the BEAT‐C study, CHWs provided 6 months of telephone‐based support to address social, practical, and emotional needs during cancer survivorship. Participants who identified social needs received weekly calls from CHWs to provide community resources and assistance in addressing needs, whereas participants without identified needs received monthly calls focused on social support and health promotion. Eligibility for the 6‐month BEAT‐C study included patients who were 6–18 months post‐diagnosis at enrollment and who self‐identified as Black. For the current qualitative study, a purposive sample of BEAT‐C participants was selected to ensure variation across hospital sites, cancer types, time since completing the intervention, time since diagnosis, and presence of identified needs. Assigned CHWs invited participants to schedule an interview with the research team. There was no relationship established between interview participants and the authors of this manuscript prior to this study. All interview participants provided verbal consent.

### 2.3 Data Collection

The study protocol was approved by the MedStar Health‐Georgetown University Institutional Review Board (IRB # 4397). Semi‐structured phone interviews were conducted between November 2024 and April 2025. The research team refined the interview guide with feedback from clinicians and qualitative doctoral level team members. Interviews were facilitated by a female research specialist with advanced training in qualitative methods and prior experience in qualitative data collection (A.M.) with supervision from a doctoral level researcher with qualitative training (H.A.). Interview questions focused on the patient's overall experience working with the CHW and receiving referrals to community organizations, factors influencing their decision to keep regular calls with the CHW, challenges and barriers they encountered in using community resources, changes in their ability to navigate resources in their community, and how discussions with the CHW and referrals to community organizations have made a difference in their QOL (see Supporting Information S1: Appendix A). The interviews lasted up to 60 minutes, and participants were compensated with a $50 check or gift card. The sample size goal was 25–35 participants, with ongoing team review to determine when thematic saturation was reached. After verbal consent, interviews were recorded and transcribed using Microsoft Teams. These recordings were transcribed verbatim and de‐identified. No additional people were present during the interview, and no repeat interviews were carried out. The research team used Otter.ai for review of transcripts and quality control. Field notes from interviews were reviewed during coding.

### 2.4 Analysis

A codebook was initially developed by a member of the research team (A.M.) to identify emergent themes using primarily an inductive approach. Subsequently, we used deductive coding to identify where themes converged with Patient‐Reported Outcomes Measurement Information System (PROMIS) social support and QOL domains [23]. The full research team then reviewed and discussed the codebook to ensure clarity and appropriateness of the codes prior to their application. Data were thematically coded using Dedoose (Version 10.0.23) by two separate reviewers (A.M., M.S.).

To ensure intercoder reliability, the two reviewers employed a consensus‐based approach. Initially, they independently coded five patient interviews, and the masked results were reviewed with a third researcher to address any discrepancies. Differences in code application and interpretation were discussed until consensus was achieved, leading to further refinement of the codebook for clarity. After finalizing the codebook, the remaining interviews were divided between the two researchers (A.M., M.S.) for independent coding. Any questions that arose were discussed between the two researchers, with additional input from a third researcher to reach a decision (H.A., M.P.C.). Emergent themes were not reviewed with participants for feedback. The research team reviewed themes and their salience (i.e., all participants, almost all, most, the majority, about half, some, few) and selected representative quotes.

## 3 Results

### 3.1 Participant Characteristics

Of 40 individuals invited to participate, 30 completed interviews, 9 were unreachable and 1 declined. Interviews lasted an average of 30 minutes (range 12–53 minutes). Participants included *n* = 14 breast, *n* = 6 prostate, *n* = 4 gastrointestinal, *n* = 4 endometrial, and *n* = 2 head and neck cancer survivors (Table 1). Median time since cancer diagnosis was 24 months (IQR = 21–31). Interviews were conducted between 2 weeks and 2 years following completion of the BEAT‐C study. Thirteen participants were diagnosed with stage II cancer, followed by stage I (*n* = 7), stage IV (*n* = 5), stage III (*n* = 2), unknown stage (*n* = 2), and stage 0 (*n* = 1). Twenty‐two participants were female and the average age was 63 years old. Participants indicated insurance from Medicaid (*n* = 11), private/commercial coverage (*n* = 6), both Medicare and Medicaid (*n* = 5), Medicare (*n* = 4), Medicare and private (*n* = 3), and Medicare, Medicaid, and private (*n* = 1). Twenty‐two (73.3%) study participants reported the presence of HRSNs, whereas *n* = 8 (26.7%) participants reported having no HRSNs.

**TABLE 1:** Participant demographics (*n* = 30).

Variables	*n*	%
Sex, female	22	73.3
Age in years, mean (SD)	63 (13)	—
Months post cancer diagnosis, median (IQR)	24 (21–31)	—
Cancer type		
Breast	14	46.7
Prostate	6	20
Head and neck	2	6.7
GI	4	13.3
Endometrial	4	13.3
Needs identified		
Yes	22	73.3
No	8	26.7
Stage		
0	1	3.3
I	7	23.3
2	13	43.3
3	2	6.7
4	5	16.7
Unknown	2	6.7
Insurance type		
Commercial/Private	6	20
Medicare	4	13.3
Medicaid	11	36.7
Medicare and private	3	10
Medicare and medicaid	5	16.7
Medicare, medicaid, and private	1	3.3

### 3.2 Qualitative Findings

Interviews highlighted several emergent themes including CHW rapport, supports provided by CHWs (emotional, instrumental, informational, and psychosocial), navigating resource referrals (communications process, barriers, facilitators, self‐efficacy), lasting effects on QOL, and recommendations for improving future social support interventions (Table 2).

**TABLE 2:** Interview themes.

Themes/Subthemes	Representative quotes
CHW rapport	“It matters how people talk to you. She made me want to join anything that she was involved with. Her personality was just one that made you feel that you could talk to her at any time. You know that customer service is so huge. Especially for me, the way people talk to me, how they respond to me, it matters. You know when you're not feeling like you are brushed off or, ‘I'm just doing my job.’ She made it very easy to want to continue. When she called, it was like, ‘Oh, hey!’ She's got that type of personality and that's huge. Yeah, that's a big factor to me.” [70‐year‐old female, endometrial cancer survivor] “She must have caught me in a very vulnerable moment, because I trusted her and I felt that I could tell her things that I couldn't tell other people. She was open with me. I talked to her about my family, and she listened, she encouraged me to keep, you know, doing whatever I needed to do.” [72‐year‐old female breast cancer survivor] “I knew she knew a lot about the things that I needed in the community. She was someone who I can talk to. Usually when you call people, you know, they hear you, but they don't listen.” [63‐year‐old female breast cancer survivor]
CHW support	
Emotional support	“She encouraged me to keep going. And a lot of times when you're dealing with a situation like I'm dealing with, you need all the encouragement you can get because sometimes you just feel like giving up. It was always good when she called in and kind of gave you a pick me up, you know, lift me up.” [72‐year‐old male prostate cancer survivor] “It was like they [patient's family] didn't want to face hearing what I was experiencing. I had no one to talk to. Those who have gone through it, I don't think that they would have a problem talking about it, but I found in my family, they just could not bear for me to discuss what I was experiencing.” [85‐year‐old female, endometrial cancer survivor] “So, I thought, for my transition after treatment that it was a beneficial experience for me, just to have someone that is checking in every so often, offering resources and then asking if I have questions about those resources or need any support. I appreciated that.” [46‐year‐old breast cancer survivor]
Instrumental support	“If I use my medical transportation, it takes 3 days, but if the doctor gets me an appointment, maybe that day or the next day, I can just call and [my CHW will] make sure I get a ride there and a ride back.” [63‐year‐old female breast cancer survivor] “She got me a Visa card and I ran out of food and I used the Visa card and got some more food… she set most of the stuff up, cause. I don't know how to do all that stuff… she's a big help.” [69‐year‐old female gastrointestinal cancer survivor] “When I had my battle and my hair was all lost, she would tell me that I can come in and they have donations for scarves and things like that. So I'll go to her if I need, like, a scarf, a different kind of scarf to cover my head with. She will always tell me to come in when they have scarves that I can come in and take a look at them and pick which ones I want.” [48‐year‐old female breast cancer survivor]
Informational support	“She just gave me some good advice about these programs and if this was something that I needed to take from it, I did. Even if I did not, she still explained it well enough. With that, maybe I can help someone else that might need those resources.” [55‐year‐old female head and neck cancer survivor] “[The CHW] was very good at letting me know if they have a community meeting or things like that, she'll send me the message, she'll text me and let me know if I have any questions and if I can get there…I did appreciate that when things were coming up, she would always reach out to me.” [48‐year‐old female breast cancer survivor] “I just mentioned because with chemo you just, okay my hair is gonna fall out because you know, that's just what it's gonna do, but I mentioned that to her and right after we got off the call, she was sending me hair loss tips, just things that I could do and manage on my own with my hair loss. I mentioned something about some food or something about trying to get grocery gift cards and she sent me information about a nutrition program. I didn't really need too much financial support with the groceries. It was more so just expensive, so I was just open to getting some assistance like that.” [37‐year‐old female breast cancer survivor] “She was really, really a very good resource and I appreciated the fact that I could call her and get my questions answered and get the information that I needed about anything related to my health for this particular situation, so I found that extremely, extremely helpful.” [81‐year‐old male prostate cancer survivor]
Psychosocial support	“Them just giving me the supportive care that I needed from them, mentally talking to me. A lot of them. I mean, I was going through a lot of hardships, during my process with this cancer and they were able to talk to me and enlighten me spiritually as well as socially how to get through those moments.” [55‐year‐old female head and neck cancer survivor] “When I was first diagnosed, I wasn't eating well, and that was kind of always stressful. So during our interview, [the CHW] would ask me, Mr. [patient's name], do you have any problem right now with stress, with this, with that? So in confidence, I will tell her, and she will tell me what to do. She'll say, Mr. [patient's name], make sure you go for your doctor's appointment. When you get to your primary physician, tell him all about this, and even your urologist, tell your urologist exactly how you feel but now I will say I'm like 75% fine, because after I went for my doctor's appointment last year, I think September or October, he said my PSA is way down. So I think [CHW] really helped me with that [communicating needs].” [55‐year‐old male prostate cancer survivor] “It was most beneficial to me in managing stress. Now, I say that because I never have been ill, and so when this is when I discovered the problem that I was having, it was, you know, I was really, sort of like really crazy, because I didn't know how to deal with it. But she helped me in terms of her calmness and her availability. I could call her at any time and to just let me know that the group was there to help, and she was there to help, and they made life so much easier. Because I have to admit, I don't know what I would have done without the transportation. I would not have known that it was available.” [81‐year‐old male prostate cancer survivor)
Navigating resource referrals	
Methods of communication for resource referral	“Anything [resources] that I had slight questions about, [the CHW] would research it and send me an e‐mail and she'd reach out to make sure I got the e‐mail.” [37‐year‐old female breast cancer survivor] “I got my resources through text and e‐mail, like they were double because I had such bad memory during that time period. My memory is now getting better, but during that time period I couldn't remember anything so she [CHW] gave me calendars, made sure that I got to my appointments on time, and helped me with resources. The [CHW] reached out to them for me…I loved that process because during times I couldn't get an appointment at a certain time, [the CHW] was able to get me an appointment earlier.” [44‐year‐old female breast cancer survivor] “When [the CHW] calls, all I have to do is tell her that I'm having a next day appointment, and she sets up everything for me right there. While we're on the telephone, she asks me if there's anything that I like or dislike about it [the transportation organization] and sends the itinerary to my phone as soon as it's time for me to come back. She answers right away and gets me a ride back home” [63‐year‐old female breast cancer survivor]
Barriers to accessing resources	
Lack of availability	“I need some mental help because I'm a little stressed out, and I do need some mental help, but she gave me a number and I called, but they never called me back to make an appointment. I Never got the appointment. It's hard, even though they tell you that you can call the number like 988 and somebody help you. I haven't been able to get through or getting nobody to respond to me. So it's not as easy as they say it is.” [77‐year‐old female breast cancer survivor] “The guy who was supposed to help with housing, he wasn't very helpful at all. Like I said, it's not [the CHW] or [the social worker], it was just him, so I would have rather have the social worker able to do all of those things with the client rather than outsource me to someone else who didn't seem to really care like that. That was the only issue I had ‘cause the guy, he just wasn't helpful at all. It was just like, oh, you need help with housing when there's no housing and just very nonchalant about it.” [44‐year‐old female breast cancer survivor]
Fees	“They're not free, and they do have costs associated with them. And we explored the free ones [community resources], and they weren't for me. They didn't meet my needs. So I just took advantage of those that I could take advantage of, but she provided me with a lot of stuff, and she probably got tired of me saying nah, that don't work for me.” [58‐year‐old female breast cancer survivor]
High burden of work	“I was connected with somebody [and they asked]: Where are you? Do you have your medication? And had I said no, the answer would have been well, here are some places that might help you with transportation. But I'm sick. You know, so giving me a list of five places to call—really, honestly, it's just a pet peeve of mine. I hope it was helpful for other people and that sort of thing is helpful because I guess that takes the legwork out of it because in some instances people don't even know where to start. So that does give them a place to start. But when you are sick, it is a lot. So yeah, perhaps a social worker, or something that can work with you or help you or something.” [58‐year‐old female breast cancer survivor]
Access delays	“They [the community organization] said you have to come [in person] first. But they don't even know if they'll see you that day that you come in. I don't like to go too far away from my house.” [77‐year‐old female breast cancer survivor]
Success stories	“[My CHW] said a car would be here in 5 minutes and it was there. When my daughter's car broke down, I had to catch a Lyft. She said, ‘He's on his way,’ and he was there. Everything, she said, it was true. And she was there, she stayed on the phone till he came with me. She didn't have to do that, but she did.” [58‐year‐old female breast cancer survivor] “I did take advantage of the food, and that was fantastic. It was good quality of food. It actually introduced me to some vegetables that I wasn't exposed prior to receiving them.” [58‐year‐old female breast cancer survivor] “[What] she gave to me was very resourceful, and she broke it down. Not just gave it to me, but she broke it down to me and explained it to me and how I can go about getting it.” [55‐year‐old female head and neck cancer survivor] “All of them [the community organizations] were pretty straightforward where they were located and everything, they were not too far from my house. My favorite part was that I was always able to get in touch with the CHW at any time.” [72‐year‐old male prostate cancer survivor] “I would say they stood out because it was just something I wasn't aware of. You kind of get a ton of generic information as a cancer patient, especially with hair loss. It was like, ‘oh, you know, I hadn't heard of this.’ And as far as the food resources, there are just tons and tons of programs in DC, but this was very specific and applicable to my needs and what I had going on.” [37‐year‐old female breast cancer survivor]
Patient self‐efficacy navigating to resources	“She gave me a little more confidence to go out and do things on my own. Like right now, I Got an issue [with rent] where they increased my money, so I just talked to them this morning. They're gonna send me paperwork where I can take care of the housing, the food stamps, and the insurance [paperwork].” [72‐year‐old female breast cancer survivor] “There's so many resources in the community too that I've never heard of that. I've learned a lot too…I think I've become much better with trying to navigate the resources and to make certain that I'm on target with what's happening through Medicare.” [81‐year‐old male prostate cancer survivor] “I'm able to obtain those resource by numbers that were given to me from her and also, I can now know how to navigate or look on Google whatever a case may be and look up some resources. If she didn't have them I could look them up now ‘cause she gave me the tools to do that.” [55‐year‐old male head and neck cancer survivor]
Lasting effects on quality of life	“So I think just because she [CHW] would ask questions about how I've been feeling, and I think that helped remind me to recognize feelings within the day that I may be experiencing, or those thoughts and it was okay to just kind of talk about it, because I just keep moving and so, some things may still impact me, but I may not always speak on it, so I think just communicating those feelings with my family or whoever has been helpful.” [46‐year‐old female breast cancer survivor] “It made a big difference because I was very, very depressed. When I met [the CHW], I feel like that my depression has lessened. Now I get out more, I move around more, I'm smiling even though I still have my issues. I took their advice when they said that I'm alive and I'm going to be okay…I make sure I take my walks just like [the CHW] told me and that really helped me” [44‐year‐old female breast cancer survivor] “I wasn't that old. I was probably 70 years old and I was functioning well. I had diabetes and a little elevated blood pressure. I was walking and doing everything just like a known person would do. And after, the treatments turned me into an invalid. When they put these bags on me, it was a challenge. When you lose your bladder and they put the bags on you, it's a big challenge. And then when they put the bags on me, they said that they could take the colostomy bag off after awhile, only to find out when they finished the surgery they couldn't because it's too long of a surgery and my age. So that means I got to carry both of the bags for the rest of my life. So that that was something that really hit me.” [72‐year‐old male prostate cancer survivor]
Recommendations for improving social support interventions	“If we started [the intervention] earlier, then we're not transitioning, because the transition part is to make you feel like ‘okay, I went through this major thing, and I'm not alone’ that to me is what it [the intervention] helped with. I'm still able to reach out, because you have all these supports and contacts going through the process. But then after, when you go back to regular life, you still need help navigating. So maybe even longer [than 6 months for the intervention], if it's going to be once we finish all of our treatment”. [46‐year‐old female breast cancer survivor] “I was diagnosed with prostate cancer and then the doctor said that if I join the [support] group, that it will help me, even though I didn't want to go, because I live in Maryland and most of the meetings are in DC, and they fall within my [work] schedule. I work in the evening, and most of the scheduled classes are in the evening, from six to eight or from five to seven. So that's why I did not join the group because they don't have anything in Maryland.” [55‐year‐old male prostate cancer survivor] “[CHWs] need to use layman's term when talking to us so people can understand in layman's term that they're helping you. A lot of times, I think it's over the person's head, they don't understand what you're saying. They just need to say that we can help you with your metro or rides to your doctor's appointment, [words] they can understand.” [57‐year‐old female endometrial cancer survivor] “If you were going to want to help people through this, you could probably put some focus on the side effects and actually talk people through those, because I think that kind of freaks you out. I can only imagine people that are going to chemotherapy, because they tell me the chemotherapy side effects are much, much worse.” [64‐year‐old male prostate cancer survivor] “I was looking for some free stuff. That's the most important thing when someone has cancer, because it's very, very expensive and you already have your expenses allocated. It doesn't even matter what insurance you have, because according to some people I have one of the best, but it's not the best because it charges me a lot of money which has caused me to need food and any free resources. Anything that I did not have to pay for, I was interested in.” [58‐year‐old female breast cancer survivor]

#### 3.2.1 CHW Rapport

Participants highlighted the unique and essential role of CHWs in their survivorship journey. CHWs developed rapport, built trust, and identified individualized supports. Almost all participants described the importance of feeling listened to with no judgment, understood, and actually cared for by the CHW, which fostered a successful relationship.

Participants reported that their CHWs felt personable, encouraging, open to talking about anything that came up in their life, and had a pleasant, warm manner. Participants valued CHWs' flexibility with call timing and consistent follow‐through with calls and providing resources. Some participants described the CHWs as knowledgeable and well‐connected to resources within the community. These CHW qualities enabled participants to open up and trust CHWs to provide them with individualized supports.

#### 3.2.2 CHW Support

##### 3.2.2.1 Emotional Support

Emotional support benefit was most reported. Every participant described enjoying having someone to talk to and appreciated knowing someone was there to check‐in on them, particularly in overcoming the emotional and psychological toll of cancer. Almost half noted the importance of open conversations about health, family, personal feelings and goals, as well as the reliability of regular check‐ins that helped them feel supported and less alone.

Encouragement was described by almost half of participants, who noted that the CHWs' positive reinforcement and motivational messages helped them stay hopeful, cope with challenges, and feel more confident in managing their health. For participants with limited existing emotional support, including those who had limited family support and those who expressed a desire to have someone to talk to outside of their family, a dependable source of empathy and understanding provided a meaningful sense of connection and reassurance. Some participants preferred having someone outside of their family to talk to, as they sought someone who would understand what they were going through and provide resources.

##### 3.2.2.2 Instrumental Support

Participants who identified social needs received both informational and instrumental support to help resolve their needs. Participants received referrals to food assistance organizations, including food banks, groceries, and tailored meal delivery. Participants received assistance in setting up transportation to medical appointments. Other participants received utility assistance, referrals to social/support groups, help with medical supplies, employment opportunities, hair loss tips, assistance with household chores, exercising opportunities, financial assistance, help with medications, rental assistance, and assistance with scheduling healthcare/dental appointments. Many of these needs were not captured on the initial screener at the beginning of the intervention but rather emerged in discussions with the CHWs over time.

Many participants reported that the resources offered had lasting effects on their daily functioning, even after completing the intervention. The ongoing supports described included transportation, food assistance, financial assistance, and help scheduling medical appointments. Two participants mentioned that the ongoing assistance with transportation to appointments has reduced their stress and anxiety about attending medical visits. Additionally, one participant reported noticeable improvements in their eating habits, including making healthier food choices, while another described becoming more consistent with daily exercise. Additionally, one participant explained that when her food deliveries stopped, she applied what she had learned about the fruits and vegetables included in the baskets and started purchasing them herself. Lastly, another participant shared that because their CHW had helped them develop the skills to engage in activities on their own, they had grown more confident.

##### 3.2.2.3 Informational Support

Participants frequently highlighted the informational support they received from their CHWs. More than half appreciated the detailed guidance on community resources, noting that CHWs helped them better understand what was available in their community. Many described their CHWs as knowledgeable and well‐connected to community resources. These qualities enabled participants to open up and trust CHWs to help provide them with individualized supports. Participants valued receiving up‐to‐date, quick access to information, and comprehensive responses to their questions and concerns. One participant noted that their CHW regularly texted them about upcoming community events. Participants frequently discussed medical questions with their CHWs, who directed them to their healthcare providers when appropriate. Even when shared resources did not feel immediately relevant, most participants still found it helpful to be aware. Specifically, many patients enjoyed receiving extensive lists of community organizations and monthly BEAT‐C newsletters featuring resources. A few participants mentioned saving this information for future use if their needs were to change.

##### 3.2.2.4 Psychosocial Support

About half of participants described the psychological impact of their illness, including depression, uncertainty, and anxiety related to their cancer prognosis. They also mentioned feeling overwhelmed by managing their illness amid competing life challenges. Some participants shared that the CHW relationship felt like confiding in a friend, providing them with a safe space to express their feelings, and even cry when needed. A few participants described turning to spirituality to cope with difficult times, with CHWs providing support by listening, which contributed to their overall psychosocial well‐being.

#### 3.2.3 Navigating Resource Referrals

##### 3.2.3.1 Methods of Communication for Resource Referrals

Participants were referred to organizations mainly through email and by phone. Other participants received referrals via text, mail, and a few participants met the CHWs in person. Support was tailored to participants' needs, ranging from providing information so they could contact organizations independently to the CHW reaching out on their behalf, with the CHW offering guidance and explanation throughout the process.

##### 3.2.3.2 Barriers to Accessing Resources

Participants described barriers with accessing community‐based organizations, including organizations being unavailable or unreachable, fees associated with the resources, high burdens to accessing the resource, and delays in services provided.

##### 3.2.3.3 Success Stories

Participants described CHW reliability in following through with what they offered. Some participants were able to improve nutrition through CHW referrals, and one participant appreciated receiving tailored information. About half of participants expressed that the referrals provided were satisfactory, useful, and helpful. Factors that facilitated participants' engagement with CBOs included ease of access and resources being geographically close to where participants lived. Flexibility and consistency emerged as two key facilitators in our study, where survivors valued the ability to call the CHW at any time should needs arise, and felt a sense of support and reassurance that someone was monitoring their wellbeing and prepared to assist if needs emerged.

##### 3.2.3.4 Patient Self‐Efficacy Navigating to Resources

Over half of participants noted increased awareness and self‐efficacy in navigating community resources. Some participants already had pre‐existing knowledge about available resources but explained that their awareness and understanding of how to access or utilize them improved after working with their CHW. Others had not been utilizing any resources at the start of the intervention and reported that the intervention broadened their understanding of what community supports were available. Participants also mentioned that their CHWs would hold them accountable to follow up with CBOs that the CHW suggested. In addition, some participants described increased confidence and ability to independently navigate resources, crediting the CHW with providing the tools and practical skills needed to find and access assistance independently. Demonstrating potential for downstream impact, several participants subsequently shared what they had learned with others going through similar challenges.

#### 3.2.4 Lasting Effects on Quality of Life

Some participants indicated cancer's long‐term impact on their physical health, including changes in diet, ability to work and exercise, and overall energy. Additionally, a few participants discussed the financial burden of cancer as a significant added expense on top of existing financial responsibilities.

When asked specifically about QOL changes resulting from CHW support, 83% (*n* = 25) of participants reported improvement. Improved mental health was reported by almost half of participants, including reductions in stress and depressive symptoms. One participant shared that the CHW helped them recognize that it was okay to have difficult thoughts and feelings, and that communicating their feelings with others can be beneficial. Some participants specifically highlighted improvements in their emotional well‐being, noting that feeling cared for and valued as a person enhanced QOL.

#### 3.2.5 Recommendations for Improving Social Support Interventions

Recommendations for improving future social support interventions included focusing on accessibility and relevance. Participants suggested that outreach information should be easy to understand across all literacy levels and that interventions should be tailored to meet specific needs of cancer survivors. They highlighted the importance of providing free resources, personalized emotional support, and managing treatment side effects. In addition, they emphasized the need for practical considerations, including greater availability of resources on weekends, extending resources beyond low‐income populations, and having geographically accessible options. Half of the participants expressed a preference for interventions longer than 6 months, valuing both the ongoing support from regular check‐ins and the additional time to better understand available resources.

## 4 Discussion

In this sample of Black cancer survivors who received 6 months of CHW support, every participant described benefit. In addition, 83% of participants reported an improvement in their QOL that persisted over time [24]. Our findings align with Uchino et al.‘s [16] theoretical model linking social support to physical health, as participants described how CHW support affected psychological and behavioral pathways perceived to influence quality of life. Participants valued CHW qualities that fostered trust, suggesting that building a relationship with their CHW over time may allow cancer survivors to feel comfortable discussing sensitive topics and expressing their emotions [25]. Additionally, CHWs with shared lived experiences may be able to form stronger connections with cancer survivors than traditional medical providers [26]. Incorporating CHWs as a part of the medical care team may offer a critical layer of support, particularly for underserved populations [27].

Our study highlights the potential of ongoing emotional support for cancer survivors. This aligns with prior studies showing that cancer survivors value having feelings acknowledged, receiving reassurance, and connecting with someone who understands them and listens [25, 26, 27]. Additionally, CHWs offered survivors an outlet to discuss their needs with someone outside their family, which was important for those who did not want to burden loved ones and preferred to speak with someone who understood what they were experiencing. Prior research has identified emotional care as a critical component of cancer experiences and outcomes and has highlighted the need for interventions that strengthen emotional support, particularly for underserved populations [26]. Our study advances this work by showing how CHWs can provide emotional support to Black cancer survivors.

Although CHWs are increasingly recognized as valuable members of care teams, their availability across U.S. health systems remains uneven, as many of these positions rely on temporary funding sources and Medicaid programs [28]. Previous research suggests that CHW interventions can improve patient outcomes and potentially reduce healthcare costs, although barriers related to funding and workforce capacity may limit their scalability [28]^.^ Assessing these barriers is important for expanding CHW‐delivered emotional support and assistance with unmet HRSNs among underserved cancer survivors. Beyond emotional support, CHWs may also play an important role in identifying and addressing practical challenges that affect survivors' QOL. Previous research using PROMIS measures found that cancer survivors often experience impaired QOL and suggested that assessing each QOL domains can help identify thresholds for intervention, prompting timely referrals and supporting more systematic management of patients' QOL trajectories [29]. However, demanding rehabilitation processes, frequent medical appointments, and negative side effects may leave cancer survivors without the mental or physical capacity to make calls, especially when they then encounter barriers within the organizations themselves. Connecting cancer survivors to community‐based organizations (CBOs) through tailored referrals can be a tangible mechanism to address unmet social needs, provide social support, and improve health outcomes [30].

Food insecurity and transportation were the most commonly reported HRSNs among participants, who stated that receiving these resources was vital to daily functioning. These findings are consistent with a prior cohort of Black cancer survivors that reported that HRSNs were associated with clinically significant differences in health‐related QOL [3]. Financial toxicity may further exacerbate these barriers, as the financial burden of cancer treatment can limit survivors' ability to afford basic necessities, including food and transportation, and negatively affect quality of life [31]. Regarding food insecurity, one prior study conducted in a general population showed that food insecurity interventions can improve health outcomes and quality of life [32]. However, few studies have examined these interventions specifically among cancer survivor populations. Participants in our study reported learning about accessible fresh fruits and vegetables. This is noteworthy given research indicating that fewer than half of cancer patients consume nutrient‐rich foods such as fruits and vegetables [33].

Access to transportation resources also had a significant effect on reducing survivors' anxiety and stress, knowing they could reliably attend healthcare appointments. This is consistent with prior research demonstrating that transportation insecurity is a major barrier to care that is associated with lower QOL, increased distress, and poorer health outcomes among cancer patients [34, 35]. Providing resource‐specific interventions consistently over a period of time rather than as a one‐time service, may have a meaningful impact on survivors' outcomes.

Given eligibility requirements and limited CBO resources, referrals alone may not fully address cancer survivors' needs. While CHWs were unable to address certain barriers such as required fees or getting to in‐person CBO appointments, CHWs could help participants identify alternative options or could assist with applications or arrange transportation. One participant appreciated that rather than only providing a CBO phone number, the CHW walked them through each step.

Further highlighting the impact of ongoing CHW contact, some participants who did not disclose any HRSNs at baseline reported emergent needs or intervention benefits over time. This may be due to limitations of the screener, new needs emerging, or increasing trust over time. Other research has also indicated that open‐ended conversations can facilitate the discussion of multiple needs, and focusing on one specific social need may often prompt the disclosure of additional needs [34].

Similar to our findings, other studies have noted CHWs can provide patients with increased awareness and self‐efficacy in navigating community resources [36, 37]. Results from a systematic evaluation showed that higher coping self‐efficacy was associated with better quality of life in cancer patients^.^ [38] Additionally, another study concluded that higher social support was associated with higher levels of self‐efficacy in cancer patients [39].

### 4.1 Clinical Implications

Our results suggest that CHWs may play an important supportive role within cancer survivorship care teams by addressing HRSNs and offering social support. Broader implementation of CHW models within oncology settings could strengthen patient‐centered care and may reduce disparities when intentionally targeted toward underserved populations with worse cancer outcomes.

### 4.2 Study Limitations

A strength of this study is that we captured rich contextual insights into how the CHW intervention affected QOL. We did not collect social equity‐related demographic data, limiting our ability to examine how these factors may have influenced participants' experiences with CHW support. However, all interviews were conducted with Black cancer survivors, focusing on a population carrying a disproportionate social needs burden and underrepresented in cancer survivorship research. While interviews were conducted between 2 weeks and 2 years after completing the intervention, raising the potential for recall bias, the extended time frame also provided an opportunity to capture both immediate and longer‐term reflections on the intervention. The sample may reflect self‐selection bias, where those who participated may represent different cancer‐related experiences than those from the larger population. However, participants who opted in likely had meaningful experiences to share, enhancing the depth of interview findings.

## 5 Conclusions

Our findings highlight that 6‐month social support from CHWs can improve emotional well‐being, self‐efficacy, and overall QOL among Black cancer survivors. Emotional support emerged as especially important, underscoring the need to address the psychological and social dimensions of survivorship alongside physical symptoms. Future research should explore opportunities to identify and address a spectrum of mental, physical, and emotional needs among cancer survivors, tailor interventions to individual circumstances, and examine the impact of such interventions on long‐term health outcomes using psychometrically validated scales, particularly among underserved populations with worse cancer outcomes. Additionally, future studies should examine implementation strategies, CHW workforce capacity, and sustainable funding models to support the integration of CHW‐led interventions into routine cancer survivorship care.

## Author Contributions

Conceptualization and protocol development: H.A., M.L.P.‐C., and A.M. Literature search: A.M. and M.S. Virtual interviews: A.M. Data analysis: A.M., M.S., and M.L.P.‐C. Initial draft preparation: A.M. and H.A. Draft review/editing: All authors.

## Conflicts of Interest

The authors declare no conflicts of interest.

## Supporting information


Supporting Information S1

## Data Availability

The data that support the findings of this study are available on request from the corresponding author. The data are not publicly available due to privacy or ethical restrictions.
