# Supplementary material for: Cancer Survivors' Experiences With Six Months of Community Health Worker Health‐Related Social Needs Support: A Qualitative Analysis of Perceived Impact
Source: Psychooncology. 2026 Nov 5;35(11):e70649. doi: 10.1002/pon.70649 (PMC13735988; doi:10.1002/pon.70649)
Supplement: Supplementary file 1 [file PON-35-e70649-s001.docx]

APPENDIX A.

**BEAT-C Post Study Interview Guide**

*Thank you for agreeing to participate in this interview regarding your care experience and discussions about social needs and community referral support. Social needs are non-medical factors that can impact health risks and outcomes, like access to food, transportation, and social support. Community referral supports are provided by organizations in your community that assist individuals in obtaining resources, like housing, food, and mental health care.*

*Your responses will be kept confidential. We will use your responses to understand how to better support patients with social needs. At any point, if you do not want to answer, we can skip the question and move to the next one.*

*With your permission, we will also be audio recording the session. This will help make sure that we do not miss anything that you say and can share with other people who are working on this project. The recording will be deleted after we have the notes transcribed and are sure we have captured all your comments accurately.*

**BEAT-C Study**

*These questions focus on your experiences during the Building Equal Access Together in Cancer (BEAT-C) study where community health workers (CHWs) call participants for 6 months to support social, practical or emotional needs (e.g., food insecurity, social support, housing instability, transportation to medical appts, employment, etc.).*

1. Can you tell me about your overall experience in the six-month study?
   a. How did your practical or emotional needs change over the course of the study *(e.g., food insecurity, social support, housing instability, transportation to medical appts, employment, etc.)*?
      i. If changes over time, please describe the needs
2. What influenced your decision to first participate in the program?
   a. Probe: What ways do you think we can encourage more people to participate?
   b. Do you think the decision to participate depended on whether people identified a social or practical need?
3. What factors influenced your decision to keep regular calls with the community health worker? (e.g., schedule, family life, medical care, etc.)
   a. In what ways do you think we could have made it easier to participate in the study?
4. Can you tell me about the community referrals provided to you through the study?
   a. How did you feel about the assistance you received?
   b. How helpful were the community resources in addressing your needs?
      i. What made them stand out to you?
      ii. How could they have been improved?
   c. Can you identify any challenges or barriers you've encountered in accessing and using community resources?
   d. In your opinion, what additional support or assistance could have been provided to make the referral process to the organizations effective in addressing your needs?
5. What organizations have the best reputation for practical, social, and emotional supports in your community?
6. What were your favorite parts about the program?
7. What were your least favorite parts about the program?

**After BEAT-C**

Now we would like to know more about your experiences after participating in the BEAT-C program.

8. Since being part of the study, what changes have you noticed in your ability to navigate resources in your community?
9. Have you continued to use the resources that were provided to you after the program?
10. In what ways do you think discussions with your community health worker about social needs and community referrals have made a difference in your quality of life, if any?
    a. Probe: Did you notice any changes in your eating, sleeping, physical activity (like walking more), going to your medical appointments, or how you are managing your everyday stress?
11. Do you think 6 months is the right length of time for the intervention?

Should be shorter / longer?
